# Prevalence and incidence of complications at diagnosis of T2DM and during follow-up by BMI and ethnicity: a matched case–control analysis

**DOI:** 10.1186/s12933-018-0712-1

**Published:** 2018-05-15

**Authors:** Ebenezer S. Owusu Adjah, Srikanth Bellary, Wasim Hanif, Kiran Patel, Kamlesh Khunti, Sanjoy K. Paul

**Affiliations:** 1 QIMR Berghofer Medical Research Institute, Brisbane, Australia; 2 Faculty of Medicine, The University of Queensland, Brisbane, Australia; 3 Aston Research Centre for Healthy Ageing, Aston University, Birmingham, UK; 4 Department of Diabetes, University Hospital Birmingham, Birmingham, UK; 5 University of Warwick, Warwick, UK; 6 Heart of England NHS Trust, Birmingham, UK; 7 Department of Health Sciences, University of Leicester, Leicester, UK; 8 Melbourne EpiCentre, University of Melbourne and Melbourne Health, Melbourne, Australia

**Keywords:** Type 2 diabetes, Body mass index, Complications, Ethnicity, Prevalence, Incidence

## Abstract

**Aims:**

To estimate the risk of developing long-term major cardiovascular and renal complications in relation to levels of body mass index (BMI) in a population of White European (WE), African-Caribbean (AC), and South Asian (SA) patients with type 2 diabetes mellitus (T2DM).

**Materials and methods:**

Patients with new diagnosis of T2DM, aged ≥ 18 years from January 2000 (n = 69,436) and their age-sex-ethnicity matched non-diabetic controls (n = 272,190) were identified from UK primary care database. Incidence rates ratios (IRRs) for non-fatal major cardiovascular events (MACE) and chronic kidney disease (CKD) in patients with T2DM compared to controls were estimated using multivariate Mantel-Cox model.

**Results:**

Among normal weight patients with T2DM, WEs had significantly higher prevalence of cardiovascular multi-morbidity (95% CI 9.5, 11.3), compared to SAs (95% CI 4.8, 9.5). AC and SA overweight and obese patients had similar prevalence, while obese WEs had significantly higher prevalence. During a median 7 years of follow-up, risk of MACE was significantly higher for overweight (95% CI of IRR 1.50, 2.46) and obese (95% CI of IRR 1.49, 2.43) SAs compared to their WE counterparts. However, similar risk levels were observed for normal weight WEs and SAs, respectively. Risk of CKD was higher and uniform for BMI ≥ 25 kg/m^2^ amongst WEs and ACs, whereas only overweight patients had significantly higher risk of CKD amongst SA [IRR 2.08 (95% CI 1.49, 2.93)].

**Conclusion:**

Risk of MACE/CKD varies over levels of BMI within each ethnic group, with overweight SAs having a disproportionate risk of CKD.

**Electronic supplementary material:**

The online version of this article (10.1186/s12933-018-0712-1) contains supplementary material, which is available to authorized users.

## Introduction

Ethnicity remains one of the key risk factors for type 2 diabetes mellitus (T2DM) and the predisposition of certain ethnic groups to develop T2DM is now well known [1]. Not only does diabetes occur early in some ethnic groups [2, 3], but there is also a greater predisposition to develop diabetes-related complications [4]. This disproportionate predisposition of certain ethnic groups to T2DM and its complications is commonly attributed to the complex interaction of genetic and environmental factors [5, 6]. Several studies have compared the prevalence and severity of diabetes complications between South Asians and White Europeans [7–12]. Although some studies have generally reported higher prevalence of some complications (particularly nephropathy and retinopathy) [11, 13], other studies have shown these differences are not as significant as thought [10, 14].

The UK Prospective Diabetes Study Group (UKPDS) evaluated the incidence of myocardial infarction (MI) by ethnicity, and found no additional risk of MI among South Asian (SA) and African-Caribbean (AC) participants, respectively compared to White European (WE) participants [10]. While this study accounted for some cardiovascular risk factors in their risk assessment model, body mass index (BMI) which is an important cardiovascular risk factor in patients with T2DM was not included. Furthermore, while other studies have evaluated the ethnicity related differences in the incidence of cardiovascular events in patients with T2DM [9, 15–17], no separate assessment of the potential differences in the risk paradigm by adiposity levels were evaluated for each ethnic group.

Given that BMI and ethnicity play important roles in cardiovascular risk profiles of patients with T2DM, we are not aware of any study that has evaluated ethnicity specific long-term cardiovascular and non-cardiovascular complications in T2DM by BMI categories at the population level. Such evaluations are of immense public health importance given the increased burden of complications associated with T2DM [18–20], and will address the knowledge gap in terms of the interplay between ethnicity, BMI, cardiovascular, and non-cardiovascular complications in patients with T2DM [21]. Therefore, the aims of this primary care based retrospective longitudinal case–control study were to evaluate (1) comorbidities and cardiovascular risk factors at diagnosis of T2DM in different ethnic groups, and (2) the likelihood of developing long term complications by BMI categories in different ethnic groups compared to non-diabetic controls.

## Methods

### Data source

Data from the primary care database of UK [The Health Improvement Network (THIN)] was used. Patients are registered with one general practitioner (GP) even though secondary care treatment can be provided elsewhere, and under terms specified by the UK’s National Health Service (NHS), GPs contribute data to THIN. Thus, daily electronic medical records (EMRs) of patients in participating practices are regularly submitted to THIN using the INPS ViSion software [22]. The database is linked to other sources of hospital and national statistics data and is demographically representative of the UK. Currently, data from over 600 general practices involved with THIN from 1990 to 2014 is available. The source population includes over 13 million patients, 85% of whom have records that are considered valid and acceptable for research. The accuracy and completeness of this database have been previously described elsewhere [23, 24]. This database provides comprehensive patient-level longitudinal information on demographic, anthropometric, clinical and laboratory measures, clinical diagnosis of diseases and events, along with complete information on prescriptions for medications with dates and doses. Clinically diagnosed diseases are recorded using Read codes [25], and with each diagnosis, an event date is entered. Similarly, prescriptions are recorded with both British National Formulary (BNF) codes and anatomical therapeutic chemical (ATC) codes along with their prescription dates.

### Study population

The primary design and results have already been published [2]. Briefly, from THIN database 69,436 patients with newly diagnosed T2DM from January 2000 were identified using a robust machine-learning algorithm, which uses the disease Read codes [25], antidiabetic medications, and lifestyle modification interventions as feeds. Patients were included if they had (1) complete information on age at diagnosis (≥ 18 years) and sex, and (2) self-identified ethnicity as WE, AC or SA. South Asians (SAs) were defined as patients with Indian, Pakistani, Sinhalese, and Bangladeshi origin, while ACs were defined as patients with Black-African and/or Caribbean origin. White Europeans (WEs) were patients with self-reported ethnicity as White, European, European, and/or New Zealand European. Those with Read codes for type 1 diabetes mellitus (T1DM) and gestational diabetes were excluded. Non-diabetic patients were patients in the THIN database with no diagnosis of any type of diabetes and had never received a prescription of an anti-diabetes therapy. Up to four non-diabetic control patients (n = 272,190) were matched to each identified T2DM patient based on age, sex and ethnicity using an exact matching algorithm. The index date for controls was defined as the date of the diabetes diagnosis for their matched cases.

### Study variables and outcome measurements

Clinical and demographic variables including smoking status, deprivation score (measure of socioeconomic status based on residential address), weight, BMI, glycated haemoglobin (HbA1c), systolic blood pressure (SBP), diastolic blood pressure (DBP), low density lipoprotein cholesterol (LDL), high density lipoprotein cholesterol (HDL), and triglycerides were extracted for each patient where appropriate. All available measures on or within 3 months prior to the index date were considered as baseline measures. For all clinical parameters, longitudinal data 12 months prior to index date and 2 years post index date were extracted on a 6-monthly window. Categories for BMI were defined following WHO established criteria as follows: normal weight (18.5–24.9 kg/m^2^), overweight (25–29.9 kg/m^2^), and obese (≥ 30 kg/m^2^). For South Asians, BMI in the ranges 18.5–22.9, 23–27.4, ≥ 27.5 kg/m^2^ were used to define normal weight, overweight and obese patients, respectively [26]. Prescription information on anti-diabetes therapies, antihypertensive agents, cardio-protective medications (CPM), weight-lowering drugs and anti-depressants were also obtained, where appropriate.

Patients with a recorded diagnosis of stroke, heart failure (HF), angina, MI, coronary artery disease (including bypass surgery and angioplasty), cancer, or renal diseases [including chronic kidney disease (CKD)] before diagnosis were considered to have relevant comorbidities at diagnosis. Subsequently, cardiovascular multi-morbidity was defined as ≥ 2 episodes of a major cardiovascular conditions at diagnosis. A composite variable for major cardiovascular events (MACE) was defined as the occurrence of non-fatal MI, HF or stroke during follow-up. Time to a specific disease event was calculated as the time from diagnosis date to the first occurrence of the disease event and patients were censored on the end date (September 2014) or on drop out date.

### Statistical analysis

Baseline characteristics of patients with incident T2DM and their matched non-diabetic controls were summarized using number (%), means (95% CI) or median (first quartile, third quartile) as appropriate. Age-sex standardized proportions of existing comorbidities at diagnosis were calculated with indirect standardisation to the internal data structure. Age groups (18–40, 41–50, 51–60, 61–70, and 71+ years) and sex (male vs. female) were used to achieve stratum-specific proportions for indirect standardisation.

Major cardiovascular event (MACE) and CKD (stage ≥ 3) incident rates (rates per 1000 person-years) were estimated by BMI categories for T2DM cases and controls separately for each ethnic group. To estimate MACE and CKD (stage ≥ 3) incidence rate ratio (IRR) for T2DM cases compared to controls, a multivariate Mantel-Cox model was fitted: adjusting for age, sex, baseline SBP, smoking status (current, ex, and never smokers), and deprivation score by stratification. Robust estimates of IRRs (95% CI) were obtained, and Bayesian information criteria (BIC) was used to compare the model fits.

## Results

### Demographic and clinical characteristics

The demographic and clinical profiles of T2DM patients (n = 69,436) and matched non-diabetic controls (n = 272,190) are presented in Table 1. Overall, the mean age at diagnosis was 57 years, 57% were male, and median follow-up time was similar across T2DM cases and their non-diabetic controls (7 years). Within subgroups defined by ethnicity, T2DM patients and their non-diabetic controls were well matched on age and sex distributions. The distribution of current or ex-smokers in T2DM patients and controls were 55 and 50%, respectively, and the proportions of patients with SBP ≥ 140 mmHg were 39 and 18%, respectively.

**Table 1:** Baseline clinical characteristics of patients with T2DM and their matched non-diabetic controls separately for each ethnic group

	White European (296,288)		African-Caribbean (16,958)		South Asian (28,380)		Overall n (341,626)	
	T2DM	Control	T2DM	Control	T2DM	Control	T2DM	Control
Patients^a^	60,233 (20)	236,055 (80)	3425 (20)	13,533 (80)	5778 (20)	22,602 (80)	69,436 (20)	272,190 (80)
Age at index (years)^b^	58 (58.2, 58.4)	58 (58.3, 58.4)	49 (48.2, 49.0)	49 (48.4, 48.8)	47 (46.3, 46.9)	47 (46.4, 46.7)	57 (56.8, 57.0)	57 (56.8, 56.9)
Age groups^a^								
18–40	4530 (8)	17,912 (8)	744 (22)	2932 (22)	1707 (30)	6685 (30)	6981 (10)	27,529 (10)
41–50	11,297 (19)	44,306 (19)	1278 (37)	5063 (37)	2097 (36)	8196 (36)	14,672 (21)	57,565 (21)
51–60	16,552 (28)	65,033 (28)	843 (25)	3326 (25)	1217 (21)	4767 (21)	18,612 (27)	73,126 (27)
61–70	17,010 (28)	66,485 (28)	415 (12)	1641 (12)	559 (10)	2185 (10)	17,984 (26)	70,311 (26)
71+	10,844 (18)	42,319 (18)	145 (4)	571 (4)	198 (3)	769 (3)	11,187 (16)	43,659 (16)
Male^a^	34,342 (57)	134,630 (57)	1778 (52)	7040 (52)	3232 (56)	1,2631 (56)	39,352 (57)	154,301 (57)
Current smokers^a^	12,830 (21)	46,926 (20)	449 (13)	1984 (15)	820 (14)	2839 (13)	14,099 (20)	51,749 (19)
Ex-smokers^a^	23,196 (39)	80,860 (34)	614 (18)	2066 (15)	756 (13)	2605 (12)	24,566 (35)	85,531 (31)
Deprivation status								
Highest affluence^a^	12,856 (21)	41,726 (18)	833 (24)	3548 (26)	1483 (26)	5586 (25)	15,172 (22)	50,860 (19)
Lowest affluence^a^	2500 (4)	11,462 (5)	457 (13)	1595 (12)	518 (9)	1989 (9)	3475 (5)	15,046 (6)
HbA_1c_ (%),^bd^	8.2 (8.2, 8.2)		9.1 (9.0, 9.2)		8.5 (8.4, 8.6)		8.3 (8.3, 8.3)	
Weight (kg)^b^	92.9 (92.8, 93.1)	78.7 (78.5, 78.8)	88.7 (87.9, 89.4)	81.6 (81.0, 82.1)	79.2 (78.7, 79.8)	72.2 (71.8, 72.5)	91.7 (91.5, 91.9)	78.3 (78.2, 78.4)
BMI (kg/m^2^)^b^	32.6 (32.6, 32.7)	27.8 (27.8, 27.8)	31.5 (31.3, 31.7)	28.2 (28.1, 28.2)	29.6 (29.5, 29.8)	26.3 (26.3, 26.4)	32.3 (32.3, 32.4)	27.7 (27.7, 27.7)
Normal weight^a^	4242 (7)	29,128 (12)	359 (11)	1174 (9)	360 (6)	1517 (7)	4961 (7)	31,819 (12)
Overweight^a^	14,446 (24)	178,671 (76)	842 (25)	10,611 (78)	1505 (26)	16,750 (74)	16,793 (24)	206,032 (76)
Obese^a^	41,545 (69)	28,256 (12)	2224 (65)	1748 (13)	3913 (68)	4335 (19)	47,682 (69)	34,339 (13)
SBP (mmHg)^b^	140 (139.7, 140)	136 (135.6, 135.8)	136 (135.7, 137.2)	133 (132.3, 133.3)	132 (131.0, 132.1)	128 (127.7, 128.5)	139 (138.9, 139.2)	135 (135, 135.2)
SBP ≥ 140 mmHg^a^	24,571 (41)	46,081 (20)	1029 (30)	1624 (12)	1302 (23)	1926 (9)	26,902 (39)	49,631 (18)
LDL (mg/dl)^b^	119 (118.6, 119)	122.3 (122.2, 122.3)	127 (126.4, 128.3)	128.7 (128.4, 128.9)	121 (120.3, 121.8)	123.9 (123.7, 124.1)	119 (119.2, 119.6)	122.7 (122.7, 122.8)
HDL (mg/dl)^b^	46 (45.6, 45.8)	55 (55.3, 55.4)	48 (47.4, 48.2)	57 (56.4, 56.6)	43 (43.1, 43.6)	51 (51.3, 51.5)	46 (45.5, 45.7)	55 (55.0,55.1)
Triglycerides (mg/dl)^c^	159 (121, 213)	115 (88, 159)	115 (81, 159)	82 (62, 115)	151 (115, 204)	115 (89, 168)	159 (115, 213)	115 (84, 159)
Comorbidities	18,014 (30)	46,449 (20)	382 (11)	963 (7)	647 (11)	1741 (8)	19,043 (27)	49,153 (18)
Cardio-protective drugs^a^								
Beta blockers	17,042 (28)	38,032 (16)	393 (12)	1041 (8)	710 (12)	1783 (8)	18,145 (26)	40,856 (15)
Calcium blockers	13,736 (23)	27,188 (12)	694 (20)	1535 (11)	673 (12)	1283 (6)	15,103 (22)	30,006 (11)
Statins	18,971 (32)	33,604 (14)	620 (18)	794 (6)	1205 (21)	1618 (7)	20,796 (30)	36,016 (13)
ACE inhibitors	16,165 (27)	27,922 (12)	532 (16)	857 (6)	793 (14)	1290 (6)	17,490 (25)	30,069 (11)
Follow-up^c^	7.0 (4, 11)	8.0 (4, 11)	7.0 (4, 10)	7.0 (4, 10)	6.0 (3, 10)	7.0 (4, 10)	7.0 (4, 11)	7.0 (4, 11)

*ACE* angiotensin-converting enzyme; *SBP* systolic blood pressure; *DBP* diastolic blood pressure; *LDL* low-density lipoprotein cholesterol; *HDL* high-density lipoprotein cholesterol; *Comorbidities* pre-existing cardiovascular (myocardial infarction, stroke, heart failure, angina, or coronary heart disease) or non-cardiovascular disease (renal diseases including chronic kidney disease, cancer, or depression) at the time of diagnosis

^a^n (%)

^b^Mean (95% CI)

^c^Median (Q1, Q3)

^d^Not presented for non-diabetic controls

Compared to WEs and ACs, SAs developed diabetes significantly earlier by (~ 10 and 2 years) and at lower BMI (3 and 2 kg/m^2^, Table 1). More SAs (66%) developed T2DM within the age of 50 years, while 27 and 59% of WEs and ACs developed the disease within the same age limit, respectively. Significantly higher proportions of WE cases and controls had SBP above 140 mmHg (41 and 21%), compared to ACs (30 and 12%) and SAs (23 and 9%), respectively.

### Prevalence of comorbidities at diagnosis

T2DM cases had a significantly higher proportion of existing comorbidities at diagnosis compared to controls (27% vs. 18%, Table 1). The prevalence (95% CI) of cardiovascular complications at diagnosis by BMI categories among patients with T2DM, separately for each ethnic group are presented in Table 2. Among normal weight patients with T2DM, WEs had significantly higher prevalence of cardiovascular multi-morbidity (prevalence 10.4%; 95% CI 9.5, 11.3), compared to SAs (prevalence 6.8%; 95% CI 4.8, 9.5), but had similar prevalence compared to ACs (prevalence; 95% CI 4.0, 10.4). African-Caribbean and SA overweight and obese patients had similar prevalence of cardiovascular multi-morbidity across all adiposity levels, while obese WEs had significantly higher risk compared to their normal weight population and also compared to other ethnic groups (Table 2).

**Table 2:** Age-sex adjusted prevalence (95% CI) of cardiovascular complications at diagnosis by BMI categories among patients with T2DM, separately for each ethnic group

	Prevalence (95% CI)			
	MACE	MI	HF	STROKE
Normal weight				
White European	10.4 (9.5, 11.3)	4.6 (4.0, 5.2)	4.6 (4.1, 5.2)	5.0 (4.4, 5.6)
African-Caribbean	6.5 (4.0, 10.4)	2.0 (0.9, 4.9)	2.1 (0.9, 4.7)	4.2 (2.3, 7.7)
South Asian	6.8 (4.8, 9.5)	4.0 (2.6, 6.2)	4.0 (2.6, 6.2)	3.1 (1.8, 5.1)
Overweight				
White European	11.7 (11.3, 12.2)	6.1 (5.7, 6.5)	6.1 (5.7, 6.5)	5.2 (4.9, 5.6)
African-Caribbean	7.2 (5.1, 9.9)	2.1 (1.0, 4.1)	2.1 (1.0, 4.1)	4.9 (3.4, 7.2)
South Asian	9.0 (7.4, 10.9)	5.3 (4.1, 6.9)	5.3 (4.1, 6.9)	3.7 (2.7, 5.1)
Obese				
White European	12.6 (12.3, 12.9)	6.5 (6.3, 6.7)	6.5 (6.3, 6.7)	5.4 (5.2, 5.6)
African-Caribbean	5.5 (4.1, 7.4)	1.1 (0.5, 2.5)	1.1 (0.5, 2.5)	4.4 (3.2, 6.2)
South Asian	8.5 (6.2, 11.7)	4.7 (2.9, 7.4)	4.7 (2.9, 7.4)	2.5 (1.5, 4.1)

**Fig. 1:**
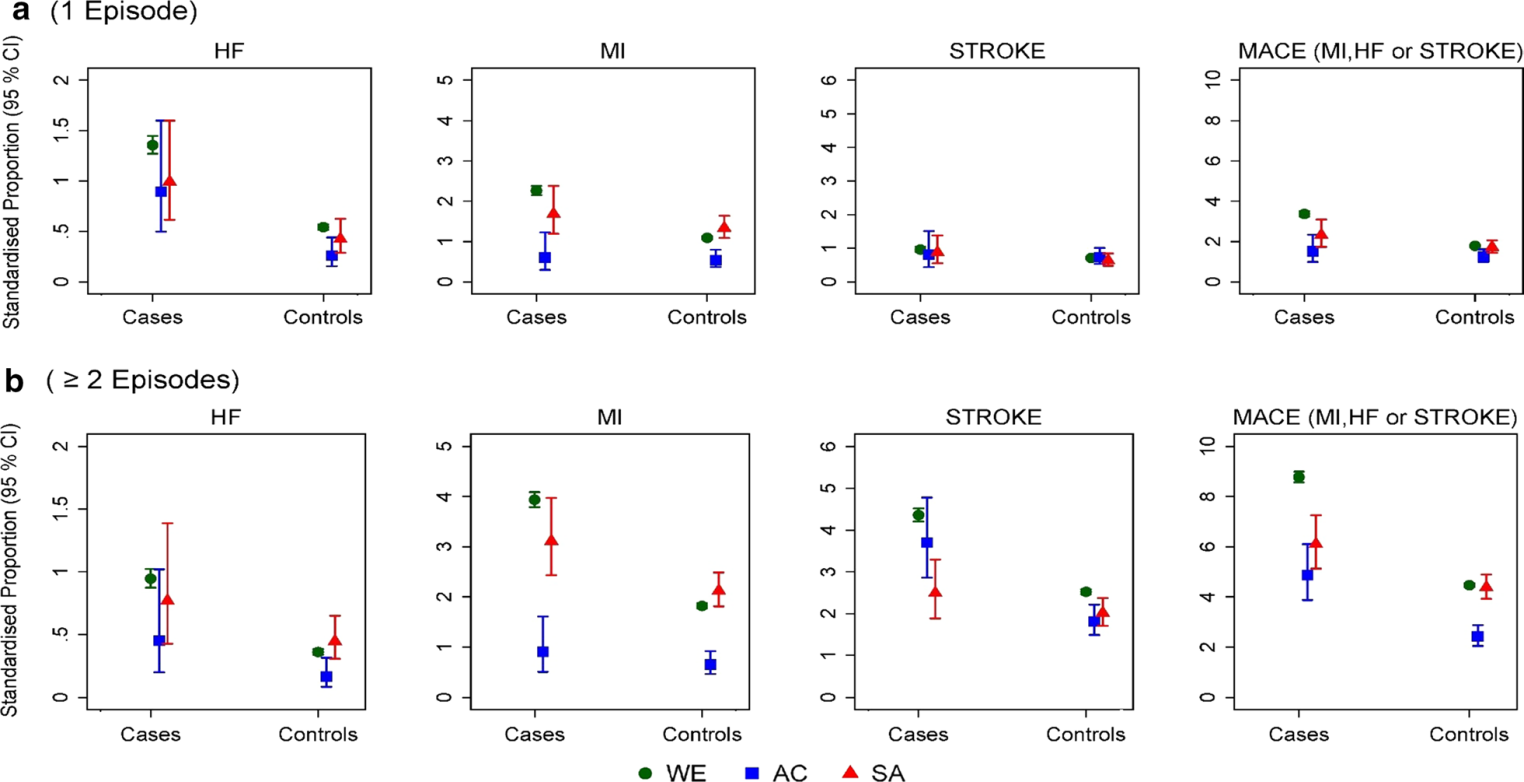
Age-sex standardised proportions [% (95 CI)] of macrovascular diseases at diagnosis for patients with T2DM and their matched controls, separately for each ethnic group. **a** The proportion of patients with at least one episode of a macrovascular event at diagnosis; **b** The proportion of patients with two or more episodes of a macrovascular disease events at diagnosis. [*HF* Heart failure; *MACE* Three (3) point major cardiovascular event defined as the occurrence of myocardial infarction, heart failure or stroke before diagnosis]. *WE* White European; *AC* African-Caribbean; *SA* South Asian

The prevalence of cardiovascular and non-cardiovascular diseases at diagnosis between T2DM cases and their non-diabetic controls, separately for each ethnic group are presented in Fig. 1 and Additional file 1: Figure S1 respectively. White Europeans with or without diabetes had significantly higher prevalence of cancer, compared to SA cases and controls (Additional file 1: Figure S1A). The prevalence of depression among WE cases and controls were significantly higher (95% CI of proportion—cases 21.8–22.5%; controls 17.3–17.5%) compared to other ethnic groups, while SA and AC cases and controls had similar prevalence (range of 95% CI of prevalence 6.6–9.7%). The prevalence of CKD at diagnosis was similar across all ethnic groups and did not differ significantly between T2DM cases and their non-diabetic controls (Additional file 1: Figure S1).

### Incidence of major cardiovascular diseases during follow-up

**Fig. 2:**
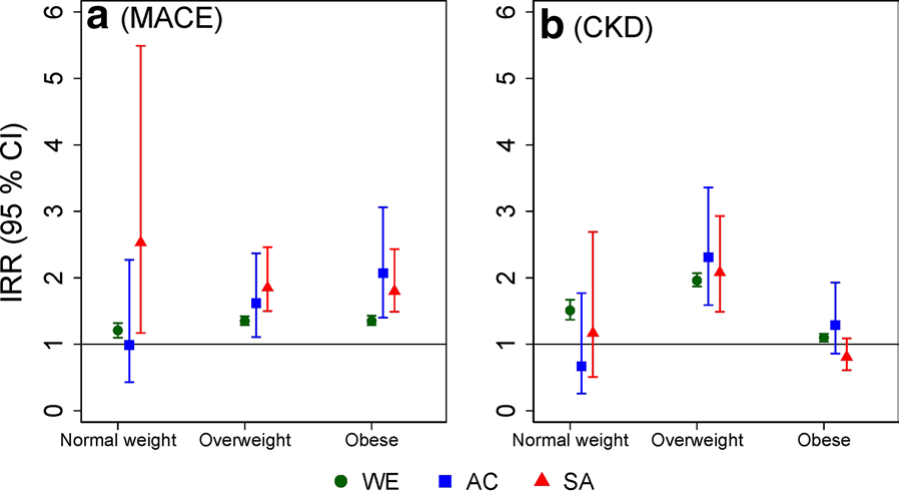
Adjusted incidence rate ratios [IRR (95% CI)] for MACE, and CKD in T2DM cases vs. matched non-diabetic controls without established comorbidities at index date. Data are presented separately by ethnicity for each BMI category at index date. *WE* White European; *AC* African-Caribbean; *SA* South Asian

In individuals without any history of comorbidities at index date, the rates per 1000 person-years and incidence rate ratios for non-fatal major cardiovascular events and chronic kidney disease during follow-up in patients with T2DM, compared to non-diabetic controls, are presented in Additional file 1: Tables S1 and S2, and Fig. 2 separately for ethnic groups and BMI categories at index date.

Overall, the risk of developing MACE in patients with T2DM, compared to non-diabetic controls, were similar for WEs (95% CI of IRR 1.29, 1.38) and ACs (95% CI of IRR 1.34, 2.25), but significantly higher for SAs (95% CI of IRR 1.56, 2.22) compared to WEs (Additional file 1: Table S1).

The risk of developing MACE was significantly higher for overweight (95% CI of IRR 1.50, 2.46) and obese (95% CI of IRR 1.49, 2.43) SAs compared to their WE counterparts (95% CI of IRR 1.29, 1.42 in overweight; 1.29, 1.43 in obese). However, similar risk levels were observed for WEs and SAs who were normal weight (Fig. 2a, Additional file 1: Table S1).

White European patients with T2DM had similar rates of MACE (range of 95% CI of rate/1000 person-years 10.55, 14.66, Additional file 1: Table S1) across all BMI level, and these rate estimates were almost two-fold higher compared to that across all adiposity levels in ACs (range of 95% CI of rate/1000 person-years 2.96, 8.78) and SAs (range of 95% CI of rate/1000 person-years 4.69, 12.91, Additional file 1: Table S1).

### Incidence of chronic kidney disease (stage 3 and above) during follow-up

Across all BMI categories, the rates of CKD were consistently higher among WE cases (range of 95% CI of IR 12.89, 19.73) and controls (range of 95% CI of IR 6.31, 8.48), compared to AC cases (range of 95% CI of IR 3.04, 10.89) and controls (range of 95% CI of IR 2.52, 7.20), and SA cases (range of 95% CI of IR 2.66, 9.21) and controls (range of 95% CI of IR 1.11, 3.54, Additional file 1: Table S2). While obese WEs with T2DM had significantly lower CKD incidence rate compared patients with BMI < 30 kg/m^2^, the observed CKD incidence rates were similar across all BMI groups in WEs without diabetes. The incidence rates for CKD were similar across all BMI categories among AC and SA cases. Obese SAs with diabetes had almost half the incidence rate for CKD (IR 3.9) compared to ACs (IR 7.3) and about one-fourth compared to WEs (IR 13.4).

The risk of developing CKD in normal weight and obese patients with T2DM, compared to non-diabetic controls, was significantly higher among WEs only (Fig. 2b). However, overweight individuals with T2DM had significantly higher and similar risk of developing CKD (range of 95% CI of IRR 1.5, 3.4), across ethnic groups (Fig. 2b, Additional file 1: Table S2).

## Discussion

This longitudinal case–control study of patients with newly diagnosed T2DM and their matched non-diabetic controls evaluated the prevalence of comorbidities at diagnosis of T2DM and the risk of developing long-term major cardiovascular and renal complications by BMI categories in different ethnic groups. There are several important findings from our study. Firstly, the relationship between obesity and risk of MACE/CKD does not appear to be linear. Secondly, at all levels of BMI, diabetes is associated with significantly greater risk of MACE. Thirdly, there are important distinctions between the ethnic groups, with South Asians showing greater susceptibility to MACE and CKD even at lower BMI levels.

Obesity is a major risk factor for T2DM and is an independent risk factor for cardiovascular disease (CVD) as well as CKD [27, 28]. Few studies, however, have explored the relationship between levels of adiposity and CVD in patients with T2DM and any underlying differences between ethnic groups given their differential susceptibility to T2DM. The large size of our cohort matched with a non-diabetic control population has allowed us to not only compare the effects of obesity on people with and without diabetes within each ethnic group but also to examine the differences between ethnic groups.

The independent effect of BMI on CVD risk has been confirmed in several population studies. Moreover, the linearity of this relationship has been shown in both Caucasian and Asian populations. In a study involving Asian population, the risk of CVD increased significantly with each 2 kg/m^2^ increase in BMI [29]. In patients with diabetes, however, this relationship is less clear and existing data suggest that the relationship may not be linear [30]. In our study, we did not find a linear relationship between BMI and CVD or between BMI and CKD. On the contrary, our data show that patients with diabetes have same or even greater degree (in the case of SAs) of risk even when they are of normal weight. The absence of this linear relationship between BMI and CVD may be due to the fact that the mechanisms by which BMI and diabetes influence CVD risk are different. Alternatively, the higher burden of other known risk factors for CVD (i.e., hypertension, dyslipidaemia and insulin resistance) seen in patients with diabetes could have a greater impact on the overall CVD risk thus mitigating the effects of obesity. In this context it is worth noting that interventions in patients with diabetes targeting weight loss have been less successful in lowering cardiovascular (CV) risk [31].

Across all ethnic groups, diabetes was associated with greater risk of MACE. This relationship did not change with levels of adiposity, except in ACs, suggesting that in some ethnic groups diabetes confers excess risk of MACE. These findings are not surprising given that patients with diabetes have a significantly greater burden of CV risk factors and are likely to be exposed to these risk factors for a much longer time. Similar trends were observed in relation to CKD, except in SAs, where the overall risk of CKD amongst diabetic and non-diabetic controls was similar in the overweight group, diabetes was associated with increased risk. Our data show that in addition to the elevated HbA1c, a greater proportion of patients with diabetes had poorly controlled blood pressure, elevated triglycerides and more likely to be obese or overweight than their non-diabetic counterparts. Despite the adverse risk profile, the use of cardio and reno-protective agents such as statins and ACE inhibitors was low suggesting there may have been opportunities for better control of risk factors. It must however, be noted that these figures date back to the year 2000 and that management of these known risk factors has improved considerably since then [32].

Although there are many common features, our data has highlighted important differences between ethnic groups. As expected, SAs were significantly younger than WEs and ACs whereas, WEs were more likely to have a diagnosis of cancer or depression and had higher systolic blood pressure levels. The overall IR for MACE and CKD was significantly greater amongst WEs compared to ACs or SAs and this risk was evenly distributed amongst all levels of adiposity in WEs. On the other hand, the risk of MACE and CKD was greater for SAs who were either normal and/or overweight when compared to WEs. We have previously shown that SAs develop diabetes much earlier and at significantly lower BMI than other ethnic groups [2]. It is possible that exposure to diabetes at a much younger age may result in adverse vascular profile which in turn influences the risk of MACE and CKD. It is well known that SAs have excess visceral adiposity which may contribute to the overall metabolic risk in this ethnic group even at lower levels of BMI. It is also possible that BMI may not be an ideal measure of adiposity in SA and other measures such as waist/hip ratio could instead be more appropriate when assessing adiposity in this ethnic group [33]. While there is a need for better understanding of the effects of adiposity on MACE/CKD in different ethnic groups, the clear message from this study is to recognise that SAs have a disproportionate risk of cardiovascular disease even at normal BMI.

Although the large multi-ethnic cohort and the availability of longitudinal data for a population sharing the same health care system have been the strengths of this study, it has some limitations. First, there were small number of events in BMI subgroups among African-Caribbean and South Asians. Second, we have in this study used BMI as a measure of obesity and it can be argued that BMI is not an ideal measure of obesity especially in certain ethnic groups such as SA. We are aware that this may have limited our ability to explore the relationship between adiposity and the risks of MACE/CKD. On the other hand, BMI is a commonly used measure of obesity and is well recorded than other measures such as waist/hip or waist/height ratios. Further, we have used ethnic-specific cut-offs for BMI [26] to provide as reliable an estimate of adiposity as possible.

Our understanding of the differences between ethnic groups towards susceptibility to diabetes has improved considerably in recent times. The findings of this study add to this knowledge and provide a greater understanding of the relationship between levels of adiposity and diabetes complications in different ethnic groups. The results of this study should enable clinicians to better diagnose and manage diabetes amongst people of different ethnicities.

## Additional file

**Additional file 1: Table S1.** Incidence rates, and adjusted incidence rate ratios (95% CI) for major cardiovascular events (myocardial infarction, heart failure or stroke) in T2DM cases and matched non-diabetic controls without established commorbidities at index date. Data are presented for all subjects, and seperately by BMI categories at index date. **Table S2.** Incidence rates, and adjusted incidence rate ratios (95% CI) for chronic kidney disease (stage ≥ 3) in T2DM cases and matched non-diabetic controls without established commorbidities at index date. Data are presented for all subjects, and seperately by BMI categories at index date. **Figure S1.** Age-sex standardised proportions [% (95 CI)] of selected non-cardiovascular diseases at diagnosis for patients with T2DM and their matched controls, separately for each ethnic group. **(A)** Proportion of patients with cancer at diagnosis; **(B)** Proportion of patients with depression at diagnosis; **(C)** Proportion of patients with CKD (stage 1 to 5) at diagnosis *CKD* Chronic kidney disease; *WE* White European; *AC* African-Caribbean; *SA* South Asian.

## Abbreviations

BMI: body mass index
WE: White European
AC: African-Caribbean
SA: South Asian
T2DM: type 2 diabetes mellitus
UK: United Kingdom
IRR: incident rate ratio
MACE: major cardiovascular event
CKD: chronic kidney disease
UKPDS: UK Prospective Diabetes Study
MI: myocardial infarction
THIN: The Health Improvement Network
GP: general practice
EMR: electronic medical records
NHS: National Health Service
BNF: British National Formulary
ATC: anatomical therapeutic chemical
T1DM: type 1 diabetes mellitus
HbA1c: glycated haemoglobin
SBP: systolic blood pressure
DBP: diastolic blood pressure
LDL: low density lipoproteins
HDL: high density lipoproteins
WHO: World Health Organisation
CPM: cardio-protective medications
HF: heart failure
