# Supplementary material for: Prevalence and incidence of complications at diagnosis of T2DM and during follow-up by BMI and ethnicity: a matched case–control analysis
Source: Cardiovasc Diabetol. 2018 May 15;17:70. doi: 10.1186/s12933-018-0712-1 (PMC5952414; doi:10.1186/s12933-018-0712-1)

**LEGEND**

**Additional Table S1**: Incidence rates, and adjusted incidence rate ratios (95% CI) for major cardiovascular events (myocardial infarction, heart failure or stroke) in T2DM cases and matched non-diabetic controls without established commorbidities at index date. Data are presented for all subjects, and seperately by BMI categories at index date.

**Additional Table S2**: Incidence rates, and adjusted incidence rate ratios (95% CI) for chronic kidney disease (stage ≥ 3) in T2DM cases and matched non-diabetic controls without established commorbidities at index date. Data are presented for all subjects, and seperately by BMI categories at index date.

**Additional Figure S1**: Age-sex standardised proportions [% (95 CI)] of selected non-cardiovascular diseases at diagnosis for patients with T2DM and their matched controls, separately for each ethnic group. **(A)** Proportion of patients with at cancer at diagnosis; **(B)** Proportion of patients with depression at diagnosis; **(C)** Proportion of patients with CKD (stage 1 to 5) at diagnosis CKD: Chronic kidney disease; WE: White European; AC: African-Caribbean; SA: South Asian.

**Additional file 1: Table S1**: Incidence rates, and adjusted incidence rate ratios (95% CI) for major cardiovascular events (myocardial infarction, heart failure or stroke) in T2DM cases and matched non-diabetic controls without established commorbidities at index date. Data are presented for all subjects, and seperately by BMI categories at index date.

|  | **T2DM** | | | **Non-diabetic controls** | | |  |
| --- | --- | --- | --- | --- | --- | --- | --- |
|  | Follow-up ^§^ | Events (%) | IR (95% CI) | Follow-up ^§^ | Events (%) | IR (95% CI) | IRR (95% CI) ^¶^ |
| **White European (WE)** | **n=42,219** | | | **n=189,606** | | |  |
| All WE | 8 (4,11) | 3378(8) | 11.24(10.87,11.63) | 8(5,11) | 10854(6) | 7.88 (7.73, 8.03) | 1.33 (1.29,1.38) |
| Normal weight | 7 (4,11) | 252(1) | 12.96 (11.46,14.66) | 7 (4, 10) | 1145(1) | 8.18 (7.72, 8.67) | 1.20 (1.10, 1.32) |
| Overweight | 8 (4,11) | 762(2) | 11.58 (10.79,12.43) | 8(5,11) | 8809(5) | 8.01 (7.85, 8.18) | 1.35 (1.29,1.42) |
| Obese | 8 (4,11) | 2364(6) | 11.00 (10.55,11.43) | 7 (4,10) | 900(<1) | 6.51 (6.10, 6.95) | 1.35 (1.29,1.43) |
| **African-Caribbean(AC)** | **n=3,043** | | | **n=12,570** | | |  |
| All AC | 7 (4,10) | 107(4) | 5.23 (4.33, 6.32) | 7 (4,10) | 238(2) | 2.82 (2.48, 3.20) | 1.74 (1.34,2.25) |
| Normal weight | 7 (3, 9) | 9(<1) | 4.57 (2.38, 8.78) | 6 (4, 9) | 24(<1) | 3.79 (2.54, 5.65) | 0.99 (0.43,2.27) |
| Overweight | 6 (3,9) | 20(1) | 4.58 (2.96, 7.10) | 7 (4,10) | 189(2) | 2.76 (2.39, 3.18) | 1.62 (1.11, 2.37) |
| Obese | 7 (4,11) | 78(3) | 5.53 (4.43, 6.90) | 6 (3, 9) | 25(<1) | 2.62 (1.77, 3.9) | 2.07 (1.40, 3.06) |
| **South Asian (SA)** | **n=5,131** | | | **n=20,861** | | |  |
| All SA | 7 (3,10) | 213(4) | 6.38 (5.58, 7.30) | 7 (4,10) | 410(2) | 2.98 (2.71, 3.28) | 1.86 (1.56, 2.22) |
| Normal weight | 6 (3, 9) | 15(<1) | 7.78 (4.69,12.91) | 6(4,10) | 30(<1) | 3.76 (2.63,5.37) | 2.53 (1.17, 5.49) |
| Overweight | 6 (3, 10) | 53(1) | 6.45 (4.93,8.44) | 7 (4,10) | 316(2) | 3.00 (2.69, 3.35) | 1.85 (1.50, 2.46) |
| Obese | 7 (4,10) | 145(3) | 6.24 (5.30,7.34) | 5 (3, 8) | 64(<1) | 2.63 (2.06, 3.36) | 1.80 (1.49, 2.43) |

§: Median (Q1,Q3), ¶: Multivariate incident rate ratios (IRRs) were adjusted for age, sex , smoking status (never,current,or ex-smoker), deprevation score (i.e lowest affluence to highest affluence), baseline systolic blood pressure. Follow-up period was from 2000 to 2014.

Three (3) point major cardiovascular event defined as occurrence of myocardial infarction or heart failure or stroke during follow-up.

IR: Incidence rates per 1000 person-years. IRR: Incidence rate ratio

**Addiitonal file 1: Table S2**: Incidence rates, and adjusted incidence rate ratios (95% CI) for chronic kidney disease (stage ≥ 3) in T2DM cases and matched non-diabetic controls without established commorbidities at index date. Data are presented for all subjects, and seperately by BMI categories at index date.

|  | **T2DM** | | | **Non-diabetic controls** | | |  |
| --- | --- | --- | --- | --- | --- | --- | --- |
|  | Follow-up ^§^ | Events (%) | IR (95% CI) | Follow-up ^§^ | Events (%) | IR (95% CI) | IRR (95% CI) ^¶^ |
| **White European (WE)** | **n=42,219** | | | **n=189,606** | | |  |
| All WE | 8 (4,11) | 4574(11) | 14.39 (13.98,14.81) | 8(5,11) | 9571(5) | 6.68 (6.55, 6.82) | 1.47 (1.42,1.52) |
| Normal weight | 7 (4,11) | 370(1) | 17.82(16.10, 19.73) | 7 (4, 10) | 1045(1) | 7.19 (6.77, 7.64) | 1.51 (1.37,1.67) |
| Overweight | 8 (4,11) | 1168(3) | 16.72(15.79, 17.71) | 8(5,11) | 7389(4) | 6.46 (6.31, 6.61) | 1.96 (1.87,2.07) |
| Obese | 8 (4,11) | 2119(5) | 13.36(12.89, 13.84) | 7 (4,10) | 1137(1) | 8.00 (7.55, 8.48) | 1.10 (1.04,1.16) |
| **African-Caribbean(AC)** | **n=3,043** | | | **n=12,570** | | |  |
| All AC | 7 (4,10) | 152(5) | 7.25 (6.18, 8.50) | 7 (4,10) | 270(2) | 3.16 (2.80,3.56) | 1.56 (1.20,2.03) |
| Normal weight | 7 (3, 9) | 11(<1) | 5.49 (3.04, 9.91) | 6 (4, 9) | 33(<1) | 5.12 (3.64, 7.20) | 0.67 (0.26,1.77) |
| Overweight | 6 (3, 9) | 35(1) | 7.82 (5.61,10.89) | 7 (4,10) | 201(2) | 2.89 (2.52, 3.32) | 2.31 (1.59,3.36) |
| Obese | 7 (4,11) | 106(3) | 7.31 (6.05, 8.85) | 6 (3, 9) | 36(<1) | 3.74 (2.70, 5.19) | 1.29 (0.86,1.93) |
| **South Asian (SA)** | **n=5,131** | | | **n=20,861** | | |  |
| All SA | 7 (3,10) | 146(3) | 4.23 (3.60,4.98) | 7 (4,10) | 317(2) | 2.27 (2.04, 2.54) | 1.17 (0.95,1.44) |
| Normal weight | 6 (3, 9) | 10 (<1) | 4.95(2.66,9.21) | 6(3,10) | 15 (<1) | 1.85(1.11,3.06) | 1.17 (0.51,2.69) |
| Overweight | 6 (3, 10) | 42 (<1) | 4.95(3.66,6.69) | 7 (3,10) | 233 (1) | 2.18(1.92,2.48) | 2.08(1.49, 2.93) |
| Obese | 7 (4,10) | 94 (<1) | 3.92(3.20,4.80) | 5 (3, 8) | 69 (<1) | 2.80(2.21,3.54) | 0.81(0.61,1.09) |

§: Median (Q1,Q3), ¶: Multivariate incident rate ratios (IRRs) were adjusted for age, sex , smoking status (never,current,or ex-smoker), deprevation score (i.e lowest affluence to highest affluence), baseline systolic blood pressure. Follow-up period was from 2000 to 2014.

IR: Incidence rates per 1000 person-years. IRR: Incidence rate ratio;

Additional file 1: Figure S1: Age-sex standardised proportions [% (95 CI)] of selected non-cardiovascular diseases at diagnosis for patients with T2DM and their matched controls, separately for each ethnic group. **(A)** Proportion of patients with at cancer at diagnosis; **(B)** Proportion of patients with depression at diagnosis; **(C)** Proportion of patients with CKD (stage 1 to 5) at diagnosis. CKD: Chronic kidney disease; WE: White European; AC: African-Caribbean; SA: South Asian.


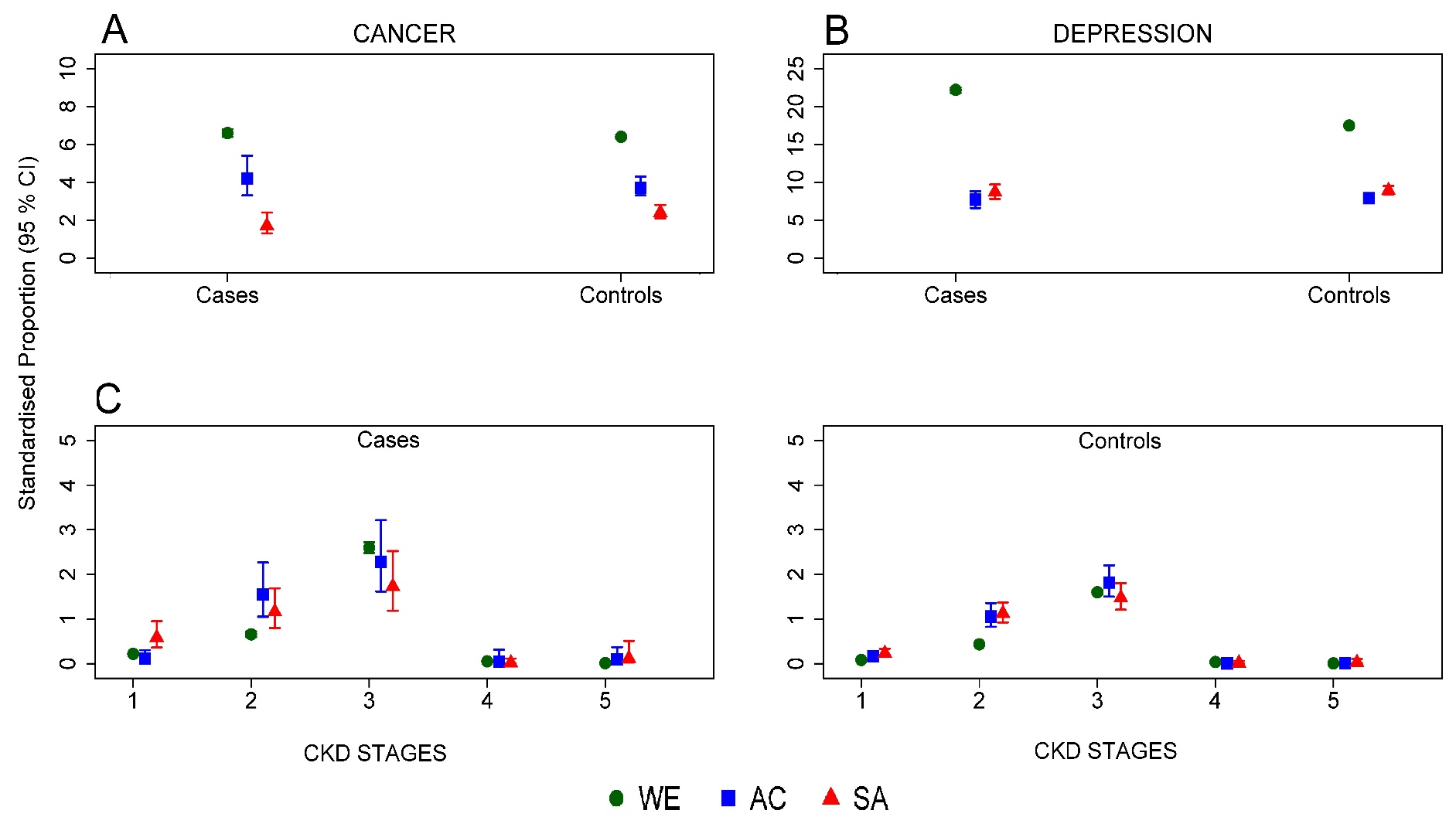

Supplement: Supplementary file 1 — Additional file 1: Table S1. Incidence rates, and adjusted incidence rate ratios (95% CI) for major cardiovascular events (myocardial infarction, heart failure or stroke) in T2DM cases and matched non-diabetic controls without established commorbidities at index date. Data are presented for all subjects, and seperately by BMI categories at index date. Table S2. Incidence rates, and adjusted incidence rate ratios (95% CI) for chronic kidney disease (stage ≥ 3) in T2DM cases and matched non-diabetic controls without established commorbidities at index date. Data are presented for all subjects, and seperately by BMI categories at index date. Figure S1. Age-sex standardised proportions [% (95 CI)] of selected non-cardiovascular diseases at diagnosis for patients with T2DM and their matched controls, separately for each ethnic group. (A) Proportion of patients with cancer at diagnosis; (B) Proportion of patients with depression at diagnosis; (C) Proportion of patients with CKD (stage 1 to 5) at diagnosis CKD Chronic kidney disease; WE White European; AC African-Caribbean; SA South Asian. [file 12933_2018_712_MOESM1_ESM.docx]
